# Caipirini: using gene sets to rank literature

**DOI:** 10.1186/1756-0381-5-1

**Published:** 2012-02-01

**Authors:** Theodoros G Soldatos, Seán I O'Donoghue, Venkata P Satagopam, Adriano Barbosa-Silva, Georgios A Pavlopoulos, Ana Carolina Wanderley-Nogueira, Nina Mota Soares-Cavalcanti, Reinhard Schneider

**Affiliations:** 1 Structural and Computational Biology Unit, European Molecular Biology Laboratory (EMBL), Heidelberg, Germany; 2 Computational Biology and Data Mining Group, Max-Delbrück Center for Molecular Medicine, Berlin, Germany; 3 Bioinformatics Graduate Program, Federal University of Paraná - UFPR (SEPT). Curitiba - PR, Brazil; 4 Departamento de Genética, Laboratório de Genética e Biotecnologia Vegetal, Centro de CiênciasBiológicas, Universidade Federal de Pernambuco, Recife, PE, Brasil; 5 ESAT-SCD/IBBT-K.U. Leuven Future Health Department, KatholiekeUniversiteit Leuven, Leuven, Belgium; 6 LIFE Biosystems GmbH, Heidelberg, Germany; 7 Garvan Institute of Medical Research, Sydney, Australia; 8 Division of Mathematics, Informatics, and Statistics, CSIRO, Sydney, Australia; 9 Luxembourg Center for Systems Biomedicine, University of Luxembourg, Luxembourg

## Abstract

**Background:**

Keeping up-to-date with bioscience literature is becoming increasingly challenging. Several recent methods help meet this challenge by allowing literature search to be launched based on lists of abstracts that the user judges to be 'interesting'. Some methods go further by allowing the user to provide a second input set of 'uninteresting' abstracts; these two input sets are then used to search and rank literature by relevance. In this work we present the service 'Caipirini' (http://caipirini.org) that also allows two input sets, but takes the novel approach of allowing ranking of literature based on one or more sets of genes.

**Results:**

To evaluate the usefulness of Caipirini, we used two test cases, one related to the human cell cycle, and a second related to disease defense mechanisms in *Arabidopsis thaliana*. In both cases, the new method achieved high precision in finding literature related to the biological mechanisms underlying the input data sets.

**Conclusions:**

To our knowledge Caipirini is the first service enabling literature search directly based on biological relevance to gene sets; thus, Caipirini gives the research community a new way to unlock hidden knowledge from gene sets derived via high-throughput experiments.

## Background

Keeping up-to-date with bioscience literature is becoming more challenging as the number of new papers appearing daily - currently over 2,000 - continues to increase. As a result, there is an increasing need for methods that can efficiently search this literature [1], and to this end a wide range of tools and services are now available [2,3]. Currently, most tools used for retrieving bioscience literature are based on keyword searches, although such approaches have limitations: firstly, it can be difficult for a researcher to find a set of keywords that exactly specify the biological functions she or he may be interested in; secondly, the ranking of results is usually not based on relevance to the biological functions of interest. Several recent methods have been proposed to address these limitations, e.g., ETBLAST [4] can launch literature searches based on a single text document such as an abstract; such methods allow searches to be defined implicitly, e.g., based on a text of interest, rather than having to explicitly define keywords. Several tools have extended this approach, allowing collections of abstracts as input, e.g., PubFinder [5] and MScanner [6].

A common problem with all literature search methods is that only a fraction of the literature retrieved is truly of interest or relevance for the end-user. Recently, a new tool, MedlineRanker [7], partly addresses this problem by allowing the end-user to define two input lists of abstracts - typically, one input list ('A') can be used for 'interesting' abstracts, and the second input list ('B') for abstracts that are 'not-interesting'. MedlineRanker then uses these two input sets to rank a third 'query' set of abstracts in order of interest, based on similarity to input sets A and B. A significant advantage of this approach is that any retrieved literature that is judged to be uninteresting can be added subsequently to the 'uninteresting' input set, and MedlineRanker can be re-run to iteratively improve the relevance of the results.

The work presented in this paper was motivated by our collaboration with a group of experimentalists interested in ranking literature corpora based on similarity to sets of genes known to be associated with specific phenotypes or conditions. For example, we were interested in ranking literature based on a set of genes known to be associated with disease resistance in the plant model organism *Arabidopsis thaliana*. In a second case, we wanted to find literature related to the S-phase of the human cell cycle, using as input one set of genes known to be involved in S-phase, and a second set of 'background' genes known to be involved with all other cell cycle phases, but not S-phase.

In searching for a tool that would suit our purposes, we surveyed existing tools for ranking and classifying biomedical literature. Many of the existing tools are based on the 'Support Vector Machine' (SVM) approach [8]; however most of these are 'hardwired' to a particular topic, e.g., alternative transcription [9], human genetic associations [10], or clinical studies [11]. Of the generic SVM tools that can be trained with any topic of interest (e.g., BibGlimpse [12]), none allow literature ranking based on gene lists, although one does precisely the opposite, namely rank genes based on literature [13]. Of the generic non-SVM tools, some can accept lists of genes as input and produce a ranked list of literature as output (e.g., Kleio [14]). However, for our purposes the existing methods have some key limitations. Firstly, users can only specify what they want - they cannot in addition specify what they do *not *want. We were interested in a method that would allow users to specify a set of literature they find uninteresting, and would use this information to improve the relevance and ranking of subsequent literature searches. A second limitation of existing methods is that they can only find literature that explicitly mentions genes in the input set. We were interested in a method that can go beyond these limitations, learn patterns in the literature associated with the input genes, and potentially return literature that may mention none of the genes in the input lists, but that discusses biological processes and functions associated with the input genes.

To meet these needs, we developed a new service ('Caipirini') that, similar to MedlineRanker, allows two main input sets, but takes the novel approach of allowing one or both input sets to be a set of gene identifiers, hence allowing literature search to be launched based on sets of genes. Caiprini also differs from similar services in that comparison of abstracts is based on keywords taken from a large dictionary of nouns and verbs. In addition, ranking in Caipirini is done using a generic SVM strategy, re-trained for each input provided by the user. Table 1 summarizes key differences between Caipirini and other comparable literature services.

**Table 1:** Tools for ranking biomedical literature using document sets

Tool	Input		Dictionary	Method
			
	Sets	Genes		
				
Kleio [14]	1	Yes	~2 million entities	VSM score
PubFinder [5]	1	No	~100,000 words from Medline	Likelihood
MScanner [6]	1	No	~25,000 MeSH terms	Bayesian
MedlineRanker [7]	1 or 2	No	Detects nouns via syntax	Bayesian
Caipirini	2	Yes	~4 million entities	SVM

## Methods

### Overview of User Workflow

On Caipirini's home page [15] the user is prompted to provide one list of input identifiers (set A). These identifiers can correspond to Entrez genes [16], Ensembl genes [17], or Medline abstracts [18] - alternatively, the user may provide a PubMed query directly as input. On the advanced version (under 'More Options') the user is able to additionally specify a second input list (set B). Sets A and B are typically used to define an 'interesting' and an 'uninteresting' (or 'background') set. When the user provides only an 'interesting' input set (set A), set B will be automatically filled with the same number of abstracts as in set A, but randomly chosen from PubMed, excluding abstracts within set A (see text below for details on how abstracts are associated with the Set A input when the user specifies genes). The third input set (set C) may contain a PubMed query or a list of PubMed identifiers - the goal of Caipirini is to rank the abstracts matching set C in order of similarity to set A (highest rank) and set B (lowest rank). When the user does not provide a set C, Caipirini will rank all abstracts in Medline (indexed by AKS2), and return those with Caiprini score over 85% (see below for details).

The classification and ranking is based on keywords extracted from abstracts associated with sets A and B. By default, Caipirini uses the following keyword types: diseases, symptoms, small molecules, genes/proteins, organisms, and other 'general biological terms'. The user can choose to deselect some or all of these types, except for the so called 'bio-terms' and 'bio-actions' (i.e., the two groups that comprise the 'general biological terms'), which are always used.

**Figure 1:**
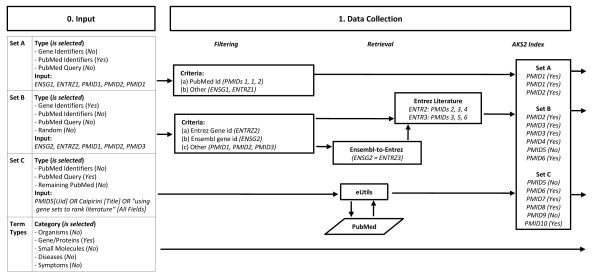
**Outline of the implemented document classification (Part I)**. The outline of the implemented methodology is demonstrated via an imaginary example: the values presented are not real; they are used just to indicate how the process works. The workflow is presented in steps: in this scenario, (*0. Input*) the user entered as input mixed identifiers from Ensembl (ENSG), Entrez (ENTRZ), and PubMed (PMID); Sets A and B consist the training set abstracts, whereas Set C is the set of abstracts to be classified; next to the standard background, only terms of type 'Gene/Protein' are selected. Then, (*1. Data Collection*) the entered data are retrieved and cleaned-up; note that in general there are Entrez identifiers that may falsely pass as PubMed identifiers (e.g., 90990), and that when there are multiple Ensembl-to-Entrez mappings for the same identifier they are all utilized - both cases not demonstrated in the example. Last, for the current version, users have suggested that multiple occurrences of entries and overlaps among sets should not be removed, e.g., for coping with imbalanced datasets. After the user has defined the input (*Step 0*) and the respective abstracts have been collected (*Step 1*), the extracted data are forwarded for further processing (*Step 2*, in Figure 2), for SVM training (*Step 3*, in Figure 3), and classification (*Step 4*, in Figure 3); finally, the results are reported to the user (*Step 5*, in Figure 3).

**Figure 2:**
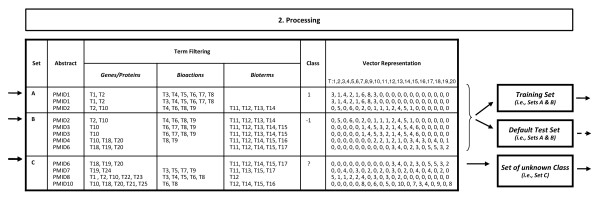
**Outline of the implemented document classification (Part II)**. The outline of the implemented methodology is demonstrated via an imaginary example (*continued*): after the input has been entered and the defined data have been collected (see Figure 1), during the next step (*2. Processing*) the abstracts indicated by the user are processed to be eventually represented in the format LIBLINEAR understands. First the terms pre-determined by the user (and the standard ones) are retrieved, as well as their frequencies in each abstract (the values presented in the figure do not represent a real scenario; they are used just to indicate how the process works) - the features used in the vectors correspond only to the selected terms found in the texts of the abstracts from sets A and B - a meta-arrangement uses vectors from sets A and B both as training set and default test set.

**Figure 3:**
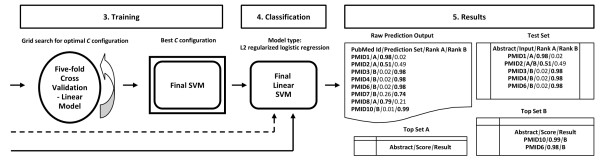
**Outline of the implemented document classification (Part III)**. The outline of the implemented methodology is demonstrated via an imaginary example (*continued*): following the initial data input and processing (see Figures 1 and 2), the next step (*3. Training*) is to find the best parameter *C *for training, based on the vectors from inputs sets A and B - once the optimal SVM configuration for the specific dataset entered by the user has been found (i.e., the value of *C *for which the highest five-fold cross validated accuracy can be achieved), it is used to create the final model. After the training, the SVM model is used to classify and rank set C, as well as the default test set (*4. Classification*). Last, result files are produced and abstracts are listed, ranked according to the predicted scores (*5. Results*).

The results comprise mainly of two lists of abstracts from set C: those found with high certainty to be more similar to set A ('Top Set A Results') and those more similar to set B ('Top Set B Results'). Nevertheless, the user has access to Caipirini's ranking scores for all abstracts. A generic outline of Caipirini's workflow is presented in Figures 1, 2 and 3, where the overview is organized in discrete steps around an imaginary example-scenario.

### Input data and initial processing

For the initial processing of input sets in Caipirini we re-used parts of the pipeline we developed for Martini [19]. However, after initial processing, the similarity with Martini ends, and Caipirini uses a very different analysis method (SVM) to achieve a very different goal (ranking of literature).

By default Caipirini assumes that sets 'A' and 'B' are lists of Entrez gene identifiers [16], in which case Caipirini retrieves, for each gene, all PubMed identifiers [18] that are referred to in the Entrez entry, including the GeneRIFs and interaction records. Caipirini also allows Ensembl gene identifiers to be used [17] - in this case, Caipirini first translates them to Entrez gene identifiers, and then to the corresponding PubMed identifiers as above; see Figure 1. The mapping between Ensembl gene, Entrez genes, and Medline abstracts is retrieved offline using SRS [20] and stored in RAM (random access memory) to enable fast access while processing jobs.

Alternatively, in the input sets 'A' and 'B', the end-user can specify either a PubMed query or a list of PubMed identifiers. For the third input set ('C'), the only formats that can be used are either a PubMed query, or PubMed identifiers. For PubMed queries, Caipirini uses Entrez Programming Utilities [21] to retrieve matching PubMed identifiers; see Figure 1. When only set 'A' is provided, a second list of PubMed identifiers (equal in size to the number of abstracts extracted from set 'A', but different from those) are randomly selected to populate set B, whereas set 'C' is considered to be all PubMed abstracts in Caipirini indexed with keywords (including abstracts from sets A and B).

The next step is to convert each PubMed identifier into a list of keywords. For this, we used an instance of AKS2, a literature analysis tool that was the engine behind the biomedical search service Novoseek. AKS2 is based on a keyword dictionary of ~3.7 million entries covering ~1.8 million genes and proteins, ~1.6 million chemicals, ~30,000 diseases, ~7,000 drugs, ~5,000 bio-actions, ~2,000 symptoms, and ~120,000 other biologically-relevant keywords. The size and breadth of the AKS2 dictionary compares favorably with similar resources such as Biothesaurus and Unified Medical Language System (UMLS), both of which have only ~2 million entries. However, our decision to use AKS2 was not based on its dictionary but on the convenience of one of its services in which the most recent half of all Medline abstracts have been pre-tagged with its dictionary, resulting in an average of 32 keywords per abstract. In addition, in previous work we found that AKS2 gave good results for the related task of keyword enrichment [19]. In the present work, we used AKS2 to construct a hash table in RAM in which each of the ~10 million Medline abstracts are linked to associated AKS2 keywords. By default, Caipirini uses all keyword types (genes, chemical, diseases, etc.) for subsequent analysis; however the web interface allows the user to exclude some types; see Figures 1 and 2.

Caipirini has a number of technical limitations: first, not all Entrez gene records have associated abstracts; second, some PubMed entries contain only titles, i.e., they have no abstract text; third, since AKS2 only indexed the most recent half of PubMed, older abstracts are not used. Finally, to reduce server load and processing time, we have limited each input field of Caipirini to a maximum number of 25,000 entries, either genes or abstracts. A job with more than this number of entries specified in any input set will not run, and the user will be asked to reduce the corresponding list's size.

### Classification of abstracts

#### Training Set

If the user has specified genes in either set A or B, these genes are first converted to a list of abstracts. Next, Caipirini creates one feature vector for each abstract: the vector has a number of dimensions equal to the total number of unique keywords found in all abstracts of sets A and B, and a length in each dimension equal to the number of occurrences of that keyword in the abstract. The same process is followed to create the vectors for set C with the difference that only terms mentioned in abstracts from the training set are taken into account; see Figure 2.

#### SVM Training

In the next step, we search for the best penalty factor *C *that gives the linear SVM model with the highest accuracy for the entered dataset - the SVM library used is LIBLINEAR [22] with the 'L2-regularized logistic regression (dual)' solver and with the tolerance termination criterion *e *set to 0.1 by default. The accuracy is assessed by LIBLINEAR using five-fold cross-validation with the training data; see Figure 3. Following the notation from [23], Caipirini does the *C *parameter search in two steps. Firstly, it evaluates the accuracy at eight points, for *c *= 2*^x^*, where *x *in [-3, 4] increased by 1.0 per step. Then, if the training set is small (i.e., smaller than 5,000 vectors), or if it is of moderate size (i.e., training vectors are less than 10,000) and the best of the tested points gives a cross-validated accuracy of less than 80%, Caipirini runs a further grid search in the neighborhood of the best *C*, see Table 2.

**Table 2:** Training the SVM: grid search for best penalty parameter *c*

*X*	*C = 2^x*	Further neighborhood search (*C *tested also for)
-3.0	0.125	0.05, 0.1, 0.15
-2.0	0.25	0.15, 0.2, 0.3, 0.35
-1.0	0.5	0.3, 0.4, 0.6, 0.7
0.0	1.0	0.75, 1.25, 1.5
1.0	2.0	1.5, 2.5, 3.0
2.0	4.0	3.0, 3.5, 4.5, 5.0, 6.0
3.0	8.0	6.0, 7.0, 9.0, 10.0, 11.0
4.0	16.0	11.0, 12.0, 13.0, 14.0, 15.0

#### Classification

The best configuration found during SVM training is used with all input data from set A and B to construct the final linear SVM model. The solver used allows assigning a 'Caipirini score' to each abstract, corresponding to the probability that the abstract belongs to either set A or B. The vectors from set C are passed into the trained model so as that each abstract is classified to belong to set A if the SVM assigns a probability of belonging to set A greater than 0.5. Vectors are assigned to set B using the opposite criteria. Finally, the abstracts from set C are listed together with their assignments, and ranked according to the Caipirini scores they received; see Figure 3.

### Performance

Many other services based on SVM are trained using pre-defined data, hence 'hardwired' to a particular topic, with only the classification done on-the-fly (e.g., GAPscreener [10]). Caipirini differs greatly in that the SVM is re-trained for each new input specified by the user. This approach has the advantage that it is likely to be more accurate; however a significant penalty is paid in the time taken to train. The exact time will depend on the size of the input sets, as well as the number of Medline abstracts per gene. As a guide to performance speed, using random sets of 25,000 abstracts in each set A and B takes about two hours to classify all PubMed abstracts indexed in Caiprini; however, running time can be highly variable, e.g., depending on unpredictable factors such as how many abstracts link to each gene, the quality of the input set, the number of keywords retrieved, the training search, and the server load.

The user can assess the reliability of Caipirini's performance in two ways. First, the Caipirini report shows a 'test set accuracy', indicating Caipirini's ability to distinguish or separate the input sets A and B. In addition, Caipirini reports a measure of the 'Cross Validation Accuracy', which is an approximate estimation of the accuracy of classification for set C; however, this estimation is based on inputs A and B, and the true classification accuracy will likely be lower, depending on what the user provides for set C. It would be possible to provide more precise estimations of the classification accuracy and significance using further re-sampling techniques, such as bootstrapping, jackknifing, or other permutation tests. However, such approaches have been developed primarily for quantitative analysis of experiment data, whereas Caipirini is more qualitative, designed to suggest which papers are interesting. Furthermore, such additional methods can be computationally expensive (e.g., [24]) and we chose not to use them, since each Caipirini analysis is already quite slow, primarily due to our use of five-fold cross-validation.

### Datasets

#### Cell-cycle datasets

To test Caipirini we used a dataset in which 158 human gene identifiers were associated with S-phase of the cell cycle, and 456 with the other three phases [19,25]. To estimate precision and accuracy, we constructed three sets of Medline abstracts using the queries specified in Table 3. The first set ('S-phase') contains abstracts that have been pre-assigned via MeSH terms [26] to S-phase specifically, and not to any of the other cell-cycle phases. This set was used to calculate true positives and false negatives. The second set ('Not S-phase') consists of Medline abstracts that have been assigned to any of the other cell cycle phases, but not to S-phase. This set was used to calculate false positives and true negatives. The final set ('Unknown phase') consists of abstracts that were related to the human cell cycle, but have not been classified by MeSH terms to any specific phase. This set was used to demonstrate the ability of Caipirini to find abstracts related to S-phase that could not be identified using MeSH terms.

**Table 3:** PubMed queries used to calculate precision and recall

Dataset	PubMed Query	Abstracts
S-phase	"humans"[MeSH Terms] AND ("S Phase"[MeSH Terms] OR "DNA Replication"[MeSH Terms]) NOT ("G1 Phase"[MeSH Terms] OR "G2 Phase"[MeSH Terms] OR "Prophase"[MeSH Terms] OR "Prometaphase"[MeSH Terms] OR "Metaphase"[MeSH Terms] OR "Anaphase"[MeSH Terms] OR "Telophase"[MeSH Terms] OR "Cytokinesis"[MeSH Terms]) AND ("2000/01/01"[PDAT]: "2008/06/31"[PDAT])	4,240
Not S-phase	"humans"[MeSH Terms] AND ("G1 Phase"[MeSH Terms] OR "G2 Phase"[MeSH Terms] OR "Prophase"[MeSH Terms] OR "Prometaphase"[MeSH Terms] OR "Metaphase"[MeSH Terms] OR "Anaphase"[MeSH Terms] OR "Telophase"[MeSH Terms] OR "Cytokinesis"[MeSH Terms]) NOT ("S Phase"[MeSH Terms] OR "DNA Replication"[MeSH Terms]) AND ("2000/01/01"[PDAT]: "2008/06/31"[PDAT])	4,329
Unknown phase	"Cell cycle" AND "humans"[MeSH Terms] NOT ("G1 Phase"[MeSH Terms] OR "G2 Phase"[MeSH Terms] OR "Prophase"[MeSH Terms] OR "Prometaphase"[MeSH Terms] OR "Metaphase"[MeSH Terms] OR "Anaphase"[MeSH Terms] OR "Telophase"[MeSH Terms] OR "Cytokinesis"[MeSH Terms] OR "S Phase"[MeSH Terms] OR "DNA Replication"[MeSH Terms]) AND ("2008/01/01"[PDAT]: "2008/06/31"[PDAT])	2,989

#### Arabidopsis datasets

We manually created a set of 90 abstracts we judged to be related to pathogen resistance in *Arabidopsis*. We also created a second set of 90 abstracts that were also related to *Arabidopsis*, but did not specifically discuss pathogen resistance mechanisms. As a test set, we defined a third set of 216 *Arabidopsis*-related abstracts for which relevance, or not, to pathogen resistance was initially unknown; this was then tested using Caipirini and subsequently verified by three curators; see Additional file 1.

## Results

### Cell-Cycle Dataset

**Figure 4:**
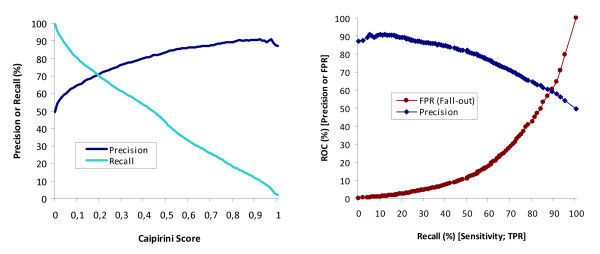
**Precision and recall values vs. Caipirini score for cell cycle dataset**. For each abstract matching the query in input set C, Caipirini calculates a score, where a value of > 0.5 means the abstract is more likely similar to set A than set B (see Methods for details). The graph on the left shows the relationship between precision, recall, and Caipirini score for the benchmark dataset, where set A are genes related to S-phase of the cell-cycle and set B are cell-cycle genes not related to S-phase. These sets were used to rank a set of abstracts already known to be related either to S-phase or to all other cell-cycle phases. With these data Caipirini achieved a moderate recall value (43% at the default Caipirini score of 0.5), indicating that just less than half of all abstracts truly related to set A were retrieved. However Caipirini achieved a good precision: 84% of abstracts with a score of > 0.5 are true positives, and higher scores give increasingly better precision. The graph on the right shows the performance of Caipirini via the relationship between precision, recall, and false positive rate for the same dataset when the threshold is set at different Caipirini scores. Note that while Caipirini's threshold increases from left to right in the Precision vs. Recall representation (left), in the ROC representations (right) the opposite is implied and Caipirini's score increases with direction from right to left.

We evaluated the performance of Caipirini using a dataset of 158 gene identifiers associated with S-phase and 456 gene identifiers associated with the other three phases of the human cell cycle (G1, G2, and M). These gene sets were then used as input to Caipirini to rank the results of a PubMed query that specifies ~4,000 abstracts known to be related to S-phase. The same input gene sets were also used to rank the results of another query specifying ~4,000 abstracts known to be related to the other cell-cycle phases, but not S-phase. Caipirini obtained a moderate recall (i.e., 43%), but had high precision (i.e., 84%); see Figure 4. That is, of all the abstracts predicted by Caipirini to be related to S-phase, 84% were correctly assigned, and Caipirini found 43% of all the abstracts known to be S-phase related. To account for the fact that some abstracts belonged both to the training and to the classified set (which can be a significant issue because the classifier gets to 'see' documents of known class and this in turn can influence performance assessment), we also calculated precision and recall after having removed the overlap (i.e., after excluding from set C abstracts from the training set): there were only minor differences observed; the recall reduced to 41% and the precision remained the same, i.e., 84%.

Another notable characteristic feature of the dataset is the 50% precision at 100% recall (see Figure 4), which implies a perfectly balanced test data set. Indeed, the two queries result in abstract lists of comparable size (see Table 3). However, such balance is unrealistic in information retrieval where 'uninteresting' is far more prevalent than 'interesting' and we used the same input gene sets to classify the results of a final query that specified a set of ~2,200 abstracts known to be related to the cell-cycle, but where the exact cell-cycle phase was not annotated with MeSH terms. For this set, we manually checked the top 20 abstracts judged by Caipirini to be most likely related to the S-phase. We found that four of these abstracts explicitly mentioned terms related to the S-phase (e.g., 'S-phase', 'DNA replication', or 'DNA repair'). The remaining sixteen abstracts did not explicitly mention processes associated with the S-phase, but they mentioned proteins and genes known to be related to the S-phase (e.g., VCP, p21, p16, Sp1, E2F, MLH1, and BRCA1) - several of them were included in the input set A.

Unlike the other two cell-cycle queries, where the correct assessment of each abstract could be determined automatically, for the third case we did not assess all abstracts since this would have required manually checking the thousands of abstracts predicted to be interesting or uninteresting, which would be quite arduous while producing results of little significance. Thus, for this dataset we did not calculate true precision or recall scores. However, this scenario (as used for the third query) is more similar to how life scientists are likely to use Caipirini, i.e., many will use queries for set C matching large numbers of abstracts, while only a small fraction of the top ranked abstracts will usually be of interest.

The three cell cycle jobs lasted 43, 45 and 39 minutes, respectively.

#### Benchmarking

Clearly similar in style, Caipirini and MedlineRanker serve similar purposes but in very distinct ways. A first noticeable difference is that MedlineRanker does not facilitate expanding a list of gene identifiers into a list of linked PubMed identifiers. Although this may be considered trivial by some, Caipirini takes this burden off the user's shoulders. Also, a user does not have to search for multiple synonyms of genes, or to disambiguate. To compare the performance of the two tools, we extracted from Caipirini the abstracts associated to each list of genes from the cell cycle dataset and used them as input to MedlineRanker (default settings applied) in order to rank the abstracts retrieved by the first two queries.

**Figure 5:**
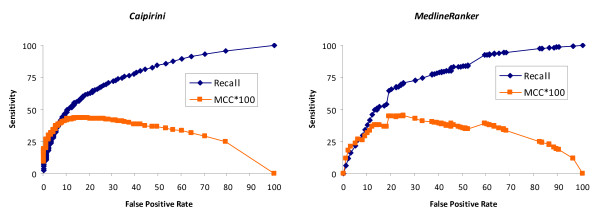
**Comparison of Caipirini with MedlineRanker in ROC space**. Using the cell cycle data set as benchmark Caipirini can be compared to other tools - When compared with MedlineRanker, the two tools performed somewhat alike, although Caipirini seems to be slightly better for this dataset. *ROC space*: recall (sensitivity) versus false positive rate for the same dataset when the threshold is set at different scores. MCC*100: to describe also with a single measure the quality of the binary (*i.e*., two-class; A vs. B) classifications we used Matthew's correlation coefficient (MCC) - Generally, it is regarded as a balanced metric that can be used even if the classes are of very different sizes. MCC values range between -1 and +1 (Coefficients valued +1, 0, and -1 represent a perfect, an average random, and an inverse prediction, respectively); in the graphs the MCC value for each score has been multiplied by one hundred.

Regarding running time, MedlineRanker was a lot faster, performing the task in only seven seconds. We also compared the accuracy of these two tools using the cell cycle dataset, and found that both performed fairly similarly (e.g., for recall 41% MedlineRanker achieves a slightly lower precision of 81%), although Caipirini seems to be slightly more robust (Figure 5). Thus, since MedlineRanker is much faster with similar performance, it is probably preferable in many cases, except where the user wants to provide gene lists as input.

### *Arabidopsis *Dataset

In addition to gene sets, Caipirini also allows sets of abstracts to be used as input. As part of an ongoing project related to disease resistance in plants [27], we tested Caipirini using sets of abstracts related to the model organism *Arabidopsis thaliana*. In this case, we used 90 abstracts known to be related to pathogen defense mechanisms in *Arabidopsis *(set A) and a further 90 abstracts also related to *Arabidopsis *but that do not discuss pathogen defense mechanisms (set B). We used Caipirini to classify 216 *Arabidopsis *abstracts (set C) that were afterwards independently checked by three of us (ABS, ACWN, and NMSC) and manually assigned as either related to pathogen defense mechanisms, or not (see Additional file 1). The criteria used included explicit mention of either disease-resistance proteins [28], of signaling hormones or other chemicals involved in disease resistance processes [29], or of processes or pathways involved in the hypersensitive response in plants [30].

Of the 216 abstracts, 122 were assigned to set A and 30 to set B by all curators, whereas for 64 there was disagreement. This spread of results underscores the importance of using several independent annotators in assessing the accuracy of a method: although the criteria for interesting vs. uninteresting abstracts may be relatively clear (as specified above), the interpretation of these criteria can vary considerably from annotator to annotator. Unlike the cell-cycle data, where the correct assessment of each abstract could be determined automatically, for the *Arabidopsis *data the 'correct' classification was considered to be that defined by the majority vote of the three independent assignments (i.e., 166 for set A and 50 for set B).

When using both sets A and B as input, Caipirini achieved 87% precision (i.e., found 188 correct assignments). Because set C is decomposed in two classes of very different sizes (dominated by set A-like abstracts), we also calculated the Matthew's correlation coefficient and found that Caipirini achieved a good prediction (with correlation equal to 0.6). Of the 24 false positives obtained, fourteen were already ambiguously categorized by manual examination (i.e., one out of the three annotators disagreed; but not the same curator every time), and of the four false negatives, two were also 'ambiguous' (i.e., one of the three annotators disagreed; the same one in both cases). When the curators later focused on the unambiguously annotated as false positive abstracts, they noted that some could be indeed considered as related to resistance, but 'indirectly' (Additional file 2).

In addition, we ran ten different jobs using the same set C but with only the 90 'interesting' *Arabidopsis *abstracts for set A, and using the random option for set B. These ten runs gave poorer performance than above, with almost all set C abstracts classified as related to set A. This illustrates the benefit of explicitly defining a background set (set B), as it can give more focused results, in this case returning a set of abstracts that almost all relate specifically to pathogen defense mechanisms in *Arabidopsis*.

Regarding speed, all eleven jobs lasted between 8 and 12 minutes each.

Both the *Arabidopsis *and cell-cycle datasets used above are available at the Caiprini web site as examples [31].

## Discussion

One of our key goals in creating Caipirini was to address the needs of experimentalists interested in using gene lists to guide literature exploration, and to find biologically relevant abstracts even if these do not explicitly mention the input genes. To our knowledge, only Caipirini and MedlineRanker enable this functionality, and Caipirini is the only method that allows the input sets to be defined using lists of genes. For example, a scientist may want to rank literature based on the difference between a set of genes associated to a primary cancer versus those associated with the metastatic form of the same cancer.

Another related goal of Caipirini - not facilitated otherwise by other tools in this way - was to allow the exploration of distinct and disjoint literature sets, gaining insight into the similar concepts they may share. For example, it would be possible to use Caipirini with the *Arabidopsis *dataset to find abstracts that discuss resistance mechanisms in other plants (see Additional file 2), effectively using knowledge from one organism to learn more about equivalent functions in similar ones. Caipirini supports such usages, particularly via the advanced options, which allow different types of keywords to be enabled or disabled - we believe that the combination of features provided here is currently not available in any other service.

In principle, methods such as Caipirini that use two input sets in this way should be capable of high precision, i.e., we would expect Caipirini to be able to find sets of abstracts with high likelihood to be related to underlying phenomena of interest. This is indeed what we see for the cell-cycle benchmark presented in Figure 4, with a precision of 84% at a Caipirini score of 0.5, and with higher Caipirini scores giving progressively better precision and fewer false positives. Thus for the biologist interested in using gene sets to search literature, our results suggest that many of the abstracts found by Caipirini are likely to correctly reflect the underlying biological difference between the two gene sets, and hence to be of interest.

Nevertheless, the performance (i.e., precision and recall) can vary greatly with different input datasets, and using Caipirini for a task may find only a moderate fraction of all relevant literature, as in the case of the cell cycle dataset. On the other hand, as shown by the *Arabidopsis *example, even in cases where the input data consists of only a small number of interesting and uninteresting cases, it can still be possible to obtain good results provided that the input is carefully selected to reflect the focused question that is asked.

Furthermore, using two input sets offers an interesting possibility to iteratively improve performance, e.g., by adding falsely classified abstracts to the 'uninteresting' input set, and re-running the classification. Such an interactive approach is currently limited slightly by the fact that Caipirini does not yet allow gene and abstract identifiers to be added to the same input set. In the near future we plan to enable such mixed input sets, and hence to further improve the ability to iteratively increase performance.

Clearly, a key limitation of Caipirini currently is its slow performance, especially for large input sets containing many well-studied genes (i.e., genes that are linked to many Medline abstracts). This is the cost paid for allowing users to train SVMs matching their particular input data every time. In the near future, we plan several changes that may improve the speed significantly. Other extensions include optional features, such as the users choosing whether they wish to be notified via e-mail when a task has finished, or whether they want statistical significance tests to be performed additionally. Furthermore, while Caiprini is currently best suited for two-set problems, we also plan to enable users to enter multiple data sets, or to save and re-use previous results in order to be able later to classify new sets of abstracts (e.g., the literature of each new month).

## Conclusions

To our knowledge Caipirini is the only service that can search literature directly based on gene sets. Though it can be slow, Caipirini allows some quite complex, new operations for extracting biological insight from gene sets. Therefore, Caipirini gives the research community a new way to unlock hidden knowledge from gene sets derived via high-throughput experiments.

### Availability and requirements

The Caipirini service is freely available at http://caipirini.org.

## Competing interests

The authors declare that they have no competing interests.

## Authors' contributions

TGS participated in the concept creation and design of the study, was the principle participant in its implementation, and further contributed to preparing the manuscript and figures. SIOD participated in the design and coordination of the study, interpretation and analysis of the results, and in preparing and proofreading the manuscript. VPS helped in the acquisition of the data, and in the administration of the system. ABS conceived and used the results of the *Arabidopsis *dataset and together with GAP, ACWN and NMSC performed manual assignments of abstracts from results. RS participated in the initial concept of the study.

## Supplementary Material

Additional file 1 **Caipirini results for the *Arabidopsis *Dataset**. The file contains (a) the annotation of the 216 abstracts of Set C (incl. some comments and notes by the curators), (b) Caipirini results from the ten runs when Set A was compared with random Sets B as background, and (c) Caipirini results for the case when Set A was tested against the manually created Set B used as reference. Click here for file

Additional file 2 **Additional discussion on the *Arabidopsis *Dataset**. Detailed explanations from the manual verification of Caipirini's results for the *Arabidopsis *data-set; 'PMID' stands for PubMed identifier. Click here for file
