# Supplementary material for: Caipirini: using gene sets to rank literature
Source: BioData Min. 2012 Feb 1;5:1. doi: 10.1186/1756-0381-5-1 (PMC3307494; doi:10.1186/1756-0381-5-1)
Supplement: Additional file 2 — Additional discussion on the Arabidopsis Dataset. Detailed explanations from the manual verification of Caipirini's results for the Arabidopsis data-set; 'PMID' stands for PubMed identifier. [file 1756-0381-5-1-S2.DOC]

**Explanation of the manual verification of Caipirini's results for the *Arabidopsis* data-set.**

(A) Among the ten non-ambiguous false positive abstracts classified by Caipirini to belong to Set A, half of them (*i.e.*, five cases) must be considered further, as follows:

These five abstracts clearly considered by the curators as not relevant to Set A (namely, PMIDs 16100230, 17012604, 18183416, 14634162 and 11577192) describe plant mechanisms in response to light. Caipirini managed via the SVM training to grasp that light is related to the context of resistance mechanisms, although by the manual evaluation the curators effectively separated light-related abstracts in Set C (*e.g.*, PMID 11959137) - which have been judged by the three curators to not be relevant - from abstracts describing the light dependency of defense responses (*e.g.*, PMID 18434604) - that have been considered as relevant. Indeed, it is known that *Arabidopsis* mechanisms are affected by the light conditions when plants are elicited by pathogens (see, PMID 15098125). Note that this last reference (*i.e.*, PMID 15098125) belonged also to Set C, and has been annotated by all three curators to be among the positive classification data set. Therefore, the five abstracts referring to light responses can 'shed some extra light' on issues regarding the overlapping mechanisms between these two distinct signaling pathways, given that such events have already been exemplified for *Arabidopsis*, as in the case of Turnip Crinkle Virus (TCV) responses conferred by the gene HRT in a light-dependent manner (see, PMID 16412080). Both of these examples can provide a biological explanation for the reasons of inclusion of light-response abstracts among the relevant classified data set.

(B) Expanding the *Arabidopsis* scenario:

Another similar observation from Set C includes a circadian rhythm-related abstract: it is known that the gene PCC1 (pathogen and circadian controlled) has its peak of expression at the end of the day and that this gene confers resistance against normally virulent oomycetes when it is over-expressed in *Arabidopsis* (see PMID 14614626) - this last reference (*i.e.*, PMID 14614626) has been correctly classified by Caipirini as relevant to Set A, agreeing with all three annotators. However, there are more abstracts in Set C which can be considered as similar to the previous cases, although in the current strict scenario they were excluded by the manual examination from being relevant to the category of Set A. In specific, among Set C abstracts two refer to salinity responses (PMIDs 16113213, and 12643272), four others refer to circadian rhythms (PMID 11463215), as well as to calcium (PMIDs 15630565 and 16072038), and to brassinosteroid metabolisms (PMID 11320207). For example, salinity is a type of abiotic stress which activates signaling pathways, such as MAPK cascades, that can likely overlap with defense responses in plants (see, PMID 11701380). Additionally, specific genes, such as the gene MBF1a of *Arabidopsis*, that have been found to confer elevated salt tolerance in transgenic plants, and, interestingly, also to confer enhanced resistance to fungal disease (see, PMID 17234157). Given that gene function overlapping seems not to be an isolated case when plants respond to environmental changes (*e.g.*, PMIDs 16927203, 16900319, and 16718483), Caipirini - when retrained with new examples - can be useful in the identification of these cases too, like before in the less indirect cases of the light and circadian rhythm abstracts.

(C) Going beyond *Arabidopsis Thaliana*:

Additional examples for such cases like in the paragraphs above include calcium (*e.g.*, see PMID 18703378) and brassinosteroid (*e.g.*, see PMID 17148785) signaling pathways that converge at some point with the disease response signaling in plants - both pathways possess genes that are implicated into disease response in plants. It has been shown that the Brassinosteroid Insensitive 1 (BRI1)-associated receptor Kinase 1 (BAK1) from *Arabidopsis thaliana*, which operates as a coreceptor of BRI1 in brassinolide (BL)-dependent plant development also plays a role in plant programmed cell death (PCD) in response to necrotrophic fungal pathogens (*e.g.*, see PMID 17583510; another reference from Set C that was correctly classified). Furthermore, a calmodulin (CaM)-related protein from tomato plays an important role in both calcium signaling and defense response. This APR134 protein from tomato, and its related protein CML43 in *Arabidopsis*, when suppressed and overexpressed in transgenic tomato and *Arabidopsis*, respectively, compromises the plant's immune response and accelerate the hypersensitive response against *Pseudomonas syringae* pv. *tomato*, respectively (see, PMID 16240180). This indicates that Caipirini can be extended further to similar literature from other organisms, too.

In conclusion, as described earlier, our results suggest that the usage of Caipirini can bring different concepts and research fields together, facilitating the understanding of several mechanisms and allowing a systems biology approach for plant sciences.
